# Outpatients satisfaction and perceptions toward pharmaceutical services in public and private hospitals in Palestine: a cross-sectional study

**DOI:** 10.1186/s40545-023-00608-2

**Published:** 2023-09-28

**Authors:** Hamzeh Al Zabadi, Renad Shraim, Raya Sawalha, Abdulsalam Alkaiyat

**Affiliations:** 1 Department of Public Health, Faculty of Medicine and Health Sciences, An-Najah National University, P.O. Box 7, Nablus, West Bank Palestine; 2 Public Health Program, Faculty of Graduate Studies, An-Najah National University, P.O. Box 7, Nablus, Palestine

**Keywords:** Hospitals, Outpatients, Perceptions, Pharmaceutical health services, Satisfaction

## Abstract

**Background:**

Pharmaceutical care is an essential component of healthcare services, and patient satisfaction with these services is crucial for improving overall health outcomes. We aimed to evaluate patient satisfaction and perception with pharmaceutical care services provided at public and private hospitals for outpatient pharmacies. This study can provide insights into the quality of pharmaceutical services provided in both settings and identify areas for improvement.

**Materials and methods:**

A cross-sectional 1-month study was conducted in three hospitals in Nablus city in the Northern District of West Bank, Palestine. Participants were a convenience sample of outpatients who attended the study-selected hospitals (two public and one private). A sample of 30 patients from each hospital was selected with a total of 90 patients. A self-administered questionnaire was used to assess socio-demographics pharmacist-related issues, waiting and working time, and medication availability.

**Results:**

A total of 90 patients were recruited. The overall level of patient satisfaction with pharmaceutical health services was moderate with a mean of 3.24 out of 5. Females represented 58.9%. The most prevalent age was (30–39) years (30%). There was a statistically significant difference in patient satisfaction with pharmaceutical services regarding working time between the morning and evening shifts (*p* value = 0.009) in favor of morning shift. No statistically significant differences in satisfaction with pharmaceutical treatments based on socio-demographics (age, gender, marital status, education level, family income, employment status, and living place), were found. Nearly, 70% of patients indicated having problems getting the medicine on their last visit to the hospital pharmacy. Only 66.7% of patients expressed satisfaction with the pharmacies’ operating (working) hours.

**Conclusions:**

Patient satisfaction with pharmaceutical care services could be enhanced by involving pharmacists in patient-oriented training and informing patients about the role of pharmacists. Patient satisfaction in the evening shift might be improved by establishing a system for continuous evaluation and improvement of pharmaceutical care services in hospitals to ensure the highest quality of care for patients in addition to implementing technology such as electronic prescribing and medication management systems that can improve the accuracy and efficiency of pharmaceutical services in hospitals.

## Background

Patient satisfaction is an essential component of healthcare service quality, it encourages patients to improve their compliance with medications and to seek healthcare service from the same healthcare provider [1–4]. Patient satisfaction should be investigated to incorporate improvements in the healthcare system [5]. However, it is based on individuals’ subjective understanding of care received [6]. Pharmaceutical services, being an integral aspect of the overall service delivery system in hospitals, play a significant role in patient satisfaction and is a key measure of hospital healthcare quality [7, 8].

Patient satisfaction regarding pharmacy services is essential for the implementation of pharmaceutical care [7], which is the provision of drug therapy by a responsible pharmacist to achieve a definite outcome to improve the patient’s quality of life, it is also the direct interaction between the pharmacist and the patient which aims to optimize the patient's health-related quality of life and achieve positive clinical outcomes within realistic economic expenditures [9, 10], rather than merely engaging pharmacists in traditional responsibilities, such as dispensing pharmaceuticals and managing drug inventory, which affects patient satisfaction and the potential role of pharmacists in improving the patient’s health [11].

Many factors influence patient satisfaction with pharmacy services, such as socio-demographic variables (age, gender, marital status, and race), waiting time, medical status, and patient expectations, pharmacy location, drug availability, and cos [6, 11–13]. Patients have favorable sentiments toward community pharmacists and ask them for guidance and medication advice [14–16], pharmacist attitude toward their patients how polite and respectful they are in addition to their respectful explanation and advice on medications management and counseling, in addition to the promptness of service and waiting time patients need to obtain the service have a great impact on patient satisfaction, the location of pharmacy and ease of patients arrival in addition to the facility working hours [6, 12, 15]. This study aimed to measure the overall patient satisfaction with pharmaceutical services provided in hospitals and its association with socio-demographic variables and focusing on Pharmacist relating issues, medications-related issues, in addition to working and waiting time-related issues. This study is significant, because it examines outpatient satisfaction and perceived attitudes about pharmaceutical treatments in public and private hospitals in Palestine. Despite the critical significance that pharmaceutical services play in healthcare outcomes, there has been little research on the subject in Palestine. As a result, this study fills a research gap and contributes to a better understanding of the pharmaceutical services landscape in the region. The study originality stems from its thorough examination of numerous aspects of pharmaceutical services and their impact on patient satisfaction. While previous studies may have analyzed specific components of pharmaceutical services, this study explores carefully many determinants, such as pharmacist–patient communication, waiting time, medication availability, working hours, and more, providing a more comprehensive picture. The findings of this study can be used to guide healthcare managers, policymakers, and researchers in enhancing patient satisfaction and the overall effectiveness of pharmaceutical services in Palestine.

## Materials and methods

### Study design, settings, and population

A cross-sectional study was conducted. In three hospitals in Nablus city in the Northern District of West Bank of Palestine (Rafedia governmental hospital, Al-Watani governmental hospital, and Al-Arabi specialized private hospital). Patients who benefit from healthcare services provided by the three pharmacies were considered as study participants.

### Study sample

The participants were a convenience sample of outpatients who attended at the study-selected hospitals. A sample of 30 patients from each hospital was selected with a total of 90 patients. Selection criteria included patients 18 years and above, attending to the hospital pharmacy for receiving pharmaceutical services. Patients with at least one visit including the present one, either in the morning or in the evening shift were included in the study.

### Data collection tool

A structured questionnaire was developed based on [5, 17]. The questionnaire comprised four parts. The first part captured the socio-demographic information on study participants. The second part consisted of 20 items measuring patient satisfaction including pharmacists-related issues using the 5-point Likert scale. The third part was used to test the satisfaction of patients with medication-related issues and this part used yes or no questions as participants perceptions. The fourth part related to waiting and working time in the pharmacy whether it is satisfactory or unsatisfactory for patients using frequency measures as perceptions of the study participants (answers: satisfactory or unsatisfactory). Several statistical tests were utilized to assess the reliability and validity of the scales used in this study. Cronbach's alpha coefficient was used to examine the reliability, which measures the consistency and reproducibility of the scales was found to be 0.82 which means a high level of internal consistency. For content and face validity, which measure the degree to which the scale covers the concept it purports to measure and the appropriateness of the items, respectively, we conducted extensive literature reviews and expert consultations.

### Ethical consideration and administrative procedures

This study was approved by the Institutional Review Board (IRB) at An-NajahNational University. An official request was submitted to hospitals’ managers and a written consent form was signed by each participant.

### Statistical analysis

The Statistical Package for Social Sciences (SPSS version 20) was used for data entry and analysis. Descriptive data were presented. Means distribution was tested for normality by Kolmogorov–Smirnov test and found to be normally distributed. One-way analysis of variance (ANOVA) was used to determine whether there were any statistically significant differences between the means of two or more independent groups and *p* value < 0.05 was always considered significant. For the 5-point Likert scale, the mean was calculated for each item for all participants and then used to interpret the results as the following: (1.80 and below considered as very low degree or poor satisfaction); (1.81–2.60 as low degree or fair satisfaction); (2.61–3.40 as moderate degree or good satisfaction); (3.41–4.20 as high degree or excellent satisfaction); and (4.21 and above as very high degree or very excellent satisfaction). We then calculated the mean of the means to estimate the overall satisfaction for the whole study population regarding the 20 items, as shown in Table 1. Furthermore, the overall degree of satisfaction for the subgroups (socio-demographic characteristics) was estimated when needed for the overall 20 items, as shown in Table 2. As only one variable showed significance in bivariate analysis, multivariate analysis was not conducted.

**Table 1:** Outpatients’ degree of satisfaction with the provided pharmaceutical health services (*N* = 90)

N	Items	Mean*	Satisfaction degree
1	The professional appearance of the pharmacy	3.01	Moderate
2	Ability of pharmacists to answer questions	3.23	Moderate
3	Pharmacist–patient relationship	3.41	High
4	Ability of pharmacists to give precautions on medicines	3.16	Moderate
5	The promptness of prescribed drugs	3.5	High
6	The professionalism of the pharmacy staff	3.47	High
7	Ability to explain the actions of medicines	3.27	Moderate
8	Ability of pharmacists to give instructions on taking medications	3.4	Moderate
9	Response of pharmacist to patients’ questions	3.38	Moderate
10	The respect shown to you by pharmacy staff	3.59	High
11	The pharmacist’s interest in your health	3.28	Moderate
12	How well pharmacists assist you in managing your medications	3.4	Moderate
13	The pharmacist’s attempt to resolve any issues you may have with your medications	3.17	Moderate
14	The pharmacists’ responsibility for your medication therapy	3.27	Moderate
15	The pharmacist’s efforts to help you improve and maintain your health	3.16	Moderate
16	The privacy of your conversations with pharmacists	2.93	Moderate
17	The pharmacist makes every effort to ensure that your medications work as they should	3.01	Moderate
18	The pharmacists’ ability to explain potential side effects	2.8	Moderate
19	Pharmacists advise you on the best way to store your medications	2.91	Moderate
20	The pharmacist gives you written instructions on how to use the medication	3.51	High
Total degree of satisfaction (mean of the means)		3.24	Moderate

*Maximum point of response (5) points (5-Likert-scale)

**Table 2:** Socio-demographic characteristics of the study participants (*N* = 90) and their degree of satisfaction with pharmaceutical services

Variable	*N* (%)	Mean	SD^#^	*P* value*
Working time				
AM	54 (60)	3.39	0.66	0.009
PM	36 (40)	3.03	0.59	
Age (year)				
18–29	23 (25.6)	3.25	0.75	0.95
30–39	27 (30)	3.3	0.64	
40–49	21 (23.3)	3.19	0.57	
50 and above	19 (21.1)	3.22	0.68	
Gender				
Male	37 (41.1)	3.36	0.74	0.16
Female	53 (58.9)	3.16	0.58	
Marital status				
Single	23 (25.6)	3.28	0.65	0.697
Married	53 (58.9)	3.2	0.68	
Divorced and widow	14 (15.6)	3.35	0.58	
Education level				
Primary education and below	14 (15.6)	3.15	0.61	0.522
Secondary education	40 (44.4)	3.33	0.72	
Bachelor and above	36 (40)	3.18	0.59	
Family income				
Less than 2000 Nis	33 (36.7)	3.24	0.73	0.262
2000–3000 Nis	32 (35.6)	3.12	0.63	
More than 3000 Nis	25 (27.8)	3.41	0.56	
Employment status				
Employee	37 (41.1)	3.32	0.69	0.346
Unemployed	53 (58.9)	3.19	0.64	
Living place				
City	34 (37.8)	3.34	0.66	0.547
Village	38 (42.2)	3.2	0.65	
Camp	18 (20)	3.15	0.66	

*Significant level for differences at (*p* < 0.05). One-way analysis of ANOVA was used for the mean difference of significance; ^#^SD: standard deviation

## Results

### Overall outpatients’ satisfaction with pharmaceutical health services

A total of 90 patients were approached and completed a self-administered questionnaire. As shown in Table 1, the overall degree of patient satisfaction with pharmaceutical health services as perceived by patients in Nablus city hospitals was found to be moderate with a mean of 3.24. The degree of satisfaction toward pharmaceutical care (respect shown to patients by pharmacists) was high as perceived by patients (3.59), and the promptness of prescription drug services was also high (3.50). The same level of satisfaction (high) of the provision of clear written instructions to patients by pharmacists (3.51) was observed. The professionalism of pharmacy staff (3.47), and patient–pharmacist relationship (3.41) were also found to be high. Table 1 reports the details.

### Outpatients’ satisfaction with pharmaceutical services by socio-demographic characteristics of the study participants

As can be seen from Table 2, the number of females was higher than males, 53 (58.9%) with the most dominant age range being (30–39) years (30%). It was noticeable that a significant proportion of the patients were living in villages accounting for 42.2%. More than half of the study participants were married with 53 participants (58.9%), 40 (44.4%) of patients’ education level was secondary school level and more than half of the participants were found to be unemployed. In addition, more than two-third of the study sample had a family income equal or less than 3000 NIS (New Israeli Shekels).

There were no statistically significant differences in satisfaction with pharmaceutical services in Nablus hospitals based on (age, gender, marital status, educational level, family income, occupation, or dwelling place). However, as shown in Table 2, there was a statistically significant difference in patient satisfaction with pharmaceutical services based on working time between the morning and evening shifts (*p* value = 0.009) in favor of the morning shift.

### Outpatients’ perceptions toward availability of medications in hospital pharmacy

Of the study participants, 70% indicated having problems getting the medicine in their last visit to the hospital pharmacy. However, only 2.2% reported having any problem with medications due to their shapes and colors. Table 3 presents the outpatients’ reported answers (yes/no) regarding the availability of medications in the hospital pharmacy.

**Table 3:** Outpatients’ perceptions toward the availability of medications in the hospital pharmacy (*N* = 90)

Medications availability aspects		Yes *N* (%)	No *N* (%)
1	Did you get this medicine the last time from the hospital pharmacy?	60 (66.6)	40 (44.4)
2	Did you have any problem to get the medicine last time?	63 (70)	27 (30)
3	Did you have any problem while taking medications due to instructions misunderstanding?	22 (24.5)	68 (75.5)
4	Was it difficult to incorporate medications into your daily life?	11 (12.2)	79 (87.8)
5	Have you experienced any problem with medications due to its shapes and color?	2 (2.2)	88 (97.8)

### Outpatients’ perceptions toward waiting and working time of the hospital pharmacy

The results shown in Table 4 revealed an important and positive influence of waiting time in pharmacy on the patients’ opinion and satisfaction. In general, almost 86.7% of the study participants reported satisfactory opinions about the average waiting time at the pharmacy to obtain their medications. However, only 66.7% of the participants reported satisfactory opinions toward the operation (working) hours of the pharmacy. More details are presented in Table 4.

**Table 4:** Outpatients satisfactory perception toward waiting and working time of the hospital pharmacy (*N* = 90)

Waiting and working time aspects of pharmaceutical services		Satisfactory *N* (%)	Unsatisfactory *N* (%)
1	Time needed to find the pharmacy	78 (86.7)	12 (13.3)
2	Time needed for registration	65 (72.2)	25 (27.8)
3	The average wait time to obtain medications at the pharmacy	78 (86.7)	12 (13.3)
4	The period of time the pharmacists offer to spend with you	84 (93.3)	6 (6.7)
5	Hours of operations of the pharmacy	60 (66.7)	30 (33.3)

## Discussion

Understanding patient satisfaction is critical as it reflects the success of a healthcare system in meeting patient expectations. In the current study, satisfaction with pharmaceutical care services was evaluated in a variety of domains, such as pharmacist interaction, waiting times, and medicine availability within the distinct cultural and socioeconomic context of Palestine.

The patient evaluation of pharmacist-related affairs was modest (3.24/5.00). This differs from studies in other countries; for example, attitudes were higher in Saudi Arabian health care settings (4.01/5.00; [12]) and (4.66/5.00, [18]). These differences indicate the impact of resources available in the respective healthcare systems, Saudi Arabia’s heavily invested system has possibly improved pharmaceutical care.

In Ethiopian public hospitals, [19] reported 2.29/5.00 overall patient satisfaction. A survey of primary healthcare centers in Brazil [6] showed that the overall percentage of patient satisfaction with pharmaceutical services was 58.4%. About pharmacist-related issues, patients indicated high satisfaction with how pharmacists show with respect to them by 3.59/5.00.

Interestingly, the patient–pharmacist relationship indicated a high satisfaction score (3.41/5.00), demonstrating the cultural value of respect in patient contacts [15]. Similarly, pharmacy staff professionalism resulted in a high satisfaction level (3.47/5.00).

Patient satisfaction was also high with the promptness of prescription drug services (3.50/5.00) and Provision of clear written instructions and counseling (3.51/5.00) this is consistent with [15] study which was conducted in Qatar.

Socio-demographic characteristics (gender, age, educational status, marital status), and independent variables were statistically not associated with patient satisfaction with pharmacy service. On the contrary in [19] study participants’ age was found to have a strong association with patient satisfaction. Patients who received pharmaceutical services in the morning shift were more satisfied than those who received services in the evening shift, presumably, because there were more pharmacists available, indicating more customized treatment. This means that the operational component of the healthcare system has an impact on patient satisfaction.

Of the study participants 70% reported having difficulty obtaining the medication during their most recent visit to the hospital pharmacy. The majority of patients reported difficulty getting drugs, indicating the Palestinian healthcare system’s financial restrictions as reported by the Palestinian Central Bureau of Statistics that the out-of-pocket household payments accounted for 39.4% of total healthcare expenditures in the Palestinian territories in 2018 [20] which is considered a significant constrain to enhance patients satisfaction in specific and the overall healthcare in general.

Out-of-pocket spending accounted for a substantial portion of total healthcare expenditures (41.8%), with the majority allocated to medicines [21]. In addition to the average waiting time at the pharmacy to acquire their drugs, study participants reported a high degree of satisfaction (86.7%) in finding the pharmacy at the healthcare institution. However, only 66.7% of participants expressed satisfaction with the pharmacy’s operating (working) hours.

These findings show how pharmacist–patient relationships, operational efficiencies, economic challenges, and cultural considerations all play important roles in determining patient satisfaction in Palestine’s unique socio-cultural and healthcare context. The findings provide useful indications for improving pharmacy services in the region.

### Limitations of study

The limitations of this study primarily revolve around the small sample size as we were only able to perform the study at three hospitals due to legislative approval concerns and due to the limited time period, we were forced to finish the data collection mission due to different organizational structures and legislative rules between three available hospitals. This limitation may affect the generalizability of our findings, as the characteristics and practices, resource availability, and organizational procedures may vary between hospitals and could influence the study findings. This study is also limited by that we did not conduct multivariate analysis. However, this was a descriptive study design aimed to evaluate satisfaction and perceptions. However, further quantitative and analytical design is necessary to find the associated factors while controlling for possible confounders. It should also be noted that in this study, as only one variable showed significance in bivariate analysis, multivariate analysis was not conducted.

## Conclusion

This study aimed to explore outpatients' satisfaction and perceptions of pharmaceutical services in public and private hospitals in Palestine. The main findings identified several elements influencing patients’ satisfaction, including clear written instructions, adequate patient–pharmacist communication, the timing of services, and the availability of medications. Socio-demographic characteristics were found to be unrelated to satisfaction levels. However, an interesting trend was identified, where patients receiving services during morning shifts reported higher satisfaction, possibly due to better pharmacy control and medication availability. These findings contribute to an improved understanding of the patient experience in hospital pharmacies and may help lead pharmacy service changes in both public and private healthcare systems. They emphasize the significance of clear communication, well-distributed operating hours, and medicine availability in increasing patient satisfaction. Future studies should include a broader range of hospitals as well as a larger sample size. Furthermore, investigating the differential impact of demographic characteristics and service timing on patient satisfaction across different contexts might give more comprehensive insights. Improved patient satisfaction can lead to better healthcare outcomes, thus knowing the elements that contribute to enhancing patient satisfaction is essential.

## Data Availability

Data are all contained within the article.
